# Adoption of pandemic treaty is historic: Compliance and accountability must now follow

**DOI:** 10.1371/journal.pgph.0004969

**Published:** 2025-08-27

**Authors:** Taran K. Deol, Elliot Hannon, Susanna Lehtimaki, Matthew M. Kavangh, Nina Schwalbe

**Affiliations:** 1 Spark Street Advisors, New York, New York, United States of America; 2 Center for Global Health Policy and Politics, Georgetown University, Washington District of Columbia, United States of America; PLOS: Public Library of Science, UNITED STATES OF AMERICA

The year 2025 began with a seismic shift in global health. In February, the United States (US) pulled out of the World Health Organization (WHO) and made clear its plan to dramatically reduce support to all areas of global public health. With US disengagement looming in the background, the adoption of the WHO Pandemic Agreement at the 78th World Health Assembly in May marked a landmark achievement, showing that with or without the US, countries were committed to a collaborative, more equitable approach to prevent, prepare for, and respond to future pandemics. The Agreement holds potential for significant change in pandemic prevention, preparedness, and response (PPR). [1] This treaty could create new international law governing the behavior of states in advance of and during pandemics. However, success hinges on implementation and accountability [1].

With 35 articles, the agreement encompasses a range of commitments, including for One Health (an approach that recognizes the interconnection between human, animal, and environmental health), research and development, technology transfer, and health system strengthening, among other areas [2]. A further annex on Pathogen Access and Benefits Sharing (PABS) will also be developed and is slated for adoption in May 2026 [1].

The agreement is ambitious, but the breadth of areas makes monitoring implementation and the associated time commitment from signatory states potentially daunting [3]. Implementation will also coincide with the implementation of the revision of the International Health Regulations (IHR), which comes into force in September 2025 [4]. This provides opportunities for synergies, given that both are binding obligations on member states.

## Defining compliance through monitoring implementation

Unlike other international treaties, the pandemic agreement is vague about compliance and accountability. The word “compliance” was negotiated out of the text, settling instead on “monitoring implementation.” But this omission doesn’t halt the act of compliance. Under international law, compliance is about whether states act in accordance with international law, whereas implementation refers to how well a norm is incorporated into domestic law or policy. Compliance is a complex process, influenced by how clearly, widely understood, and prominent the expectations are for proper state behaviour [5].

Currently, the agreement only requires “periodic reporting” by State Parties. The development of a mechanism to facilitate and strengthen implementation, including monitoring, is left to the Conference of the Parties (COP). The COP, the agreement’s governing body, comes into force a year after 60 countries ratify. To prepare for the COP, Member States have tasked an Intergovernmental Working Group (IGWG) to put forward a proposal on “the information, frequency and format of the reports” [2].

## Tools to monitor implementation

A recent review of practice indicates that treaty implementation should be monitored through a combination of self-reporting and independent review [6]. For PPR, most tools have been voluntary or self-reporting. The self-reporting States Parties’ Self-Assessment Annual Report - the primary IHR monitoring mechanism - is routinely challenged for reliability and validity, given its susceptibility to manipulation [7]. While the Joint External Evaluations were introduced to address this, it is a voluntary exercise where application remains limited [7]. The Universal Health and Preparedness Review - a pilot peer-review mechanism coordinated by the WHO - aims to foster accountability and transparency among Member States [8]. However, this undertaking is also voluntary.

The implementation of preparedness and compliance measures when public health emergencies were declared in the past was imperfect, as some states refused to engage in collective action on issues ranging from sharing data to sharing life-saving medical countermeasures, including diagnostics, drugs and vaccines. A core goal of international law is to set norms and secure coordinated behavior of states, particularly when it is not in their short-term self-interest [9]. International relations research indicates that even for “soft” international law, such as the new agreement, various mechanisms have been effective in securing compliance. These mechanisms include establishing clear norms and expected actions, opportunities for non-state actors to review progress, and channels for states to address disputes [10].

## Getting a head start on implementation, compliance, and monitoring

Although the agreement may be a few years away from coming into force, implementation, compliance, and monitoring can commence now, so that states are ready to fulfill their obligations. As what doesn’t get monitored doesn’t get done, Member States need to start to develop a monitoring framework now.

**Fig 1:**
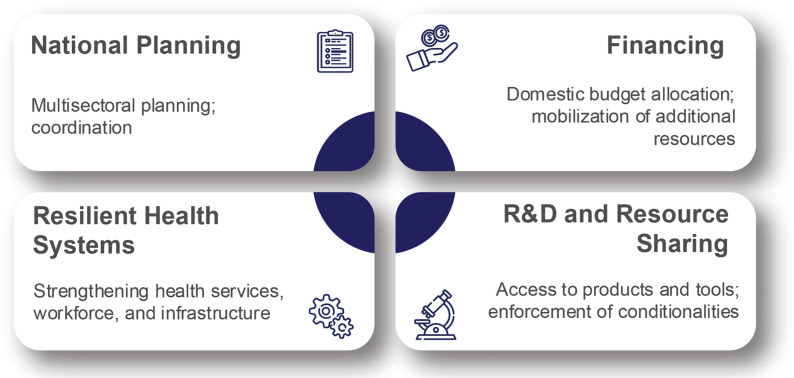
Montioring framework for the pandemic agreement-thematic areas and examples.

This framework should be a living tool with adaptive capacity to evolve with political and institutional developments. [11] It can be structured around four thematic areas core to success (Fig 1): (i) National Planning, (ii) Financing, (iii) Resilient Health Systems, (iv) Research and Development & Resource Sharing. These themes encompass the range of provisions outlined in the treaty text [11]. Recognizing the diversity of actors involved in implementation, a “draft zero” should outline both obligations at the country level and activities for the COP, IGWG, and WHO as the agreement’s Secretariat [11].

## Balancing aspiration and realism

While the road to implementation is long, developing a monitoring framework is pivotal to its success [9]. With mechanisms to support implementation, this new agreement creates an opportunity for improvement. This framework should strike a balance between tracking measurable steps and broader aspirations, between being comprehensive yet not cumbersome, and being aspirational yet realistic. It should also rely on existing indicators and align with IHR monitoring to avoid being burdensome for countries.

Learnings from other successful monitoring mechanisms, including the United Nations Framework Convention on Climate Change, the International Labor Organization, and the Intergovernmental Panel on Climate Change, demonstrate the importance of independent monitoring [6]. These experiences collectively underscore that it is never too soon to begin monitoring. What the Pandemic Agreement delivers, not what is promised, will define its legacy. And this delivery is incumbent on an effective, independent, and inclusive way to measure what has been done.
